# Suppressing Viscous Fingering in Porous Media with Wetting Gradient

**DOI:** 10.3390/ma16072601

**Published:** 2023-03-24

**Authors:** Xiongsheng Wang, Cuicui Yin, Juan Wang, Kaihong Zheng, Zhengrong Zhang, Zhuo Tian, Yongnan Xiong

**Affiliations:** 1 School of Materials and Energy, Guangdong University of Technology, Guangzhou 510006, China; 2 Guangdong Provincial Key Laboratory of Metal Toughening Technology and Application, National Engineering Research Center of Powder Metallurgy of Titanium & Rare Metals, Institute of New Materials, Guangdong Academy of Sciences, Guangzhou 510651, China

**Keywords:** wetting gradient, suppressed viscous fingering, porous media, multiphase flow, immiscible fluid displacement, lattice Boltzmann simulation

## Abstract

The viscous fingering phenomenon often occurs when a low-viscosity fluid displaces a high-viscosity fluid in a homogeneous porous media, which is an undesirable displacement process in many engineering applications. The influence of wetting gradient on this process has been studied over a wide range of capillary numbers (7.5 × 10^−6^ to 1.8 × 10^−4^), viscosity ratios (0.0025 to 0.04), and porosities (0.48 to 0.68), employing the lattice Boltzmann method. Our results demonstrate that the flow front stability can be improved by the gradual increase in wettability of the porous media. When the capillary number is less than 3.5 × 10^−5^, the viscous fingering can be successfully suppressed and the transition from unstable to stable displacement can be achieved by the wetting gradient. Moreover, under the conditions of high viscosity ratio (*M* > 0.01) and large porosity (*Φ* > 0.58), wetting gradient improves the stability of the flow front more significantly.

## 1. Introduction

The displacement of immiscible two-phase fluids in porous media is an important subject in natural and engineering fields, including oil exploration [1], carbon dioxide storage [2], fuel cells [3,4], batteries [5], water purification [6], general electrochemical energy storage [7,8] etc. For example, in oil exploration, the preferential flow of displacement fluids will bypass larger areas of oil, trapping some of the oil in the reservoir, thus reducing oil recovery efficiency [9]. In terms of carbon dioxide storage, supercritical carbon dioxide is injected into a deep saline aquifer. The viscous stability of the primary drainage process is of major interest for the injection of carbon dioxide in saline aquifers, since it determines the spread of the carbon dioxide plume in the target aquifer and consequently the initial utilization of the pore space for carbon dioxide storage [10]. It is of great significance for understanding the flow laws of immiscible fluids in porous media.

In order to better study the flow law of fluid in porous media, it is necessary to quantitatively analyze the results. There are several parameters for quantitative analysis, including the displacement efficiency, saturation, interface length and fractal dimension. The displacement efficiency refers to the percentage of the volume of the displaced fluid flowing out and the initial filling volume [11]. The saturation refers to the percentage of the displacement fluid volume in the total pore volume [12]. The saturation can be the percentage at different times, which is different from displacement efficiency. The length of the interface is defined as the length of the interface between the displacing fluid and the displaced fluid. It is often employed to characterize the instability of the fluid front. The longer the interface length, the more unstable the flow along the front of the fluid. The fractal dimension is described as a measure of the space-filling capacity of a pattern, which is used as an indicator to represent the complex geometric form, to compare the changes of details in the pattern, and to reflect the effectiveness of complex shapes occupying space. It is a measure of the irregularity of complex shapes. The more complex the fractal dimension is, the more complex the shape is [13].

The flow of fluids in porous media can be divided into three types: stable displacement, capillary fingering and viscous fingering [13]. There exist two dimensionless parameters by which to characterize the flow of fluids in porous media, including the viscosity ratio *M* = *μ*_1_/*μ*_2_ and the capillary number *Ca* = *μ*_1_*U*/*σ*; here, *μ*_1_ is the viscosity of the displacing fluid. *μ*_2_ is the viscosity of the displaced fluid, *U* is the rate at which the displaced fluid is injected, and *σ* is the surface tension. The phase diagrams of *M* and *Ca* have been summarized [14], which is of great significance for further understanding the flow laws of fluids in porous media. Zhang [15] and Zheng [16] have also summarized the relevant phase diagrams when they study the flow of fluids in porous media. The phase diagrams summarized by these researchers are not identical in the boundary ranges of different flow patterns, which may be due to the wettability, gravity and surface roughness [17].

Zhao et al. [18] studied the influence of wetting angle between fluid and porous media, and found that the influence of wettability is not monotonic. Jung et al. [19] studied the influence of wettability by combining experiments and simulations. Their research shows that wettability has a great impact on the displacement process of immiscible fluid. When the wetting angle between the displacement fluid and the porous medium is less than 80°, the saturation of the final displacement fluid is higher because the interface of the fluid front moves smoothly and the displacement fluid is not trapped. Even when studying the influence of wettability, the law of influence on wettability is not completely consistent. On the one hand, the pore channels in porous media are uneven, especially in the displacement problem in porous media in the engineering field, where the pores are very irregular. Even in the displacement problem in homogeneous porous media, the nonuniformity of the flow front interface will also have a great impact [20]. Golmohammadi et al. [21] used experimental methods to study the comprehensive effect of gravity and wettability on fluid flow in two-dimensional porous media, and found that gravity plays an important role in improving the filling effect. Hu et al. [22] studied the influence of roughness on the flow pattern in rock fractures and quantized the energy dissipated in the process of fluid invasion dominated by capillary force. Moreover, scholars have also made some progress in improving the stability of the fluid front flow in porous media. Rabbani et al. [11] proposed a method of suppressing viscous fingering by designing the gradual and monotonic variation of pore sizes along the front path. Lu et al. [23] investigated the influence of pore size gradient and pore-scale disorder on the displacement process when a non-wetting fluid displaces a wetting fluid, and found that a sufficiently large gradient can completely suppress capillary fingering.

With the development of science and technology, the investigation of immiscible fluid flow in porous media is not limited to experiments, and many scholars have also tried to use numerical simulation methods to carry out corresponding research. The lattice Boltzmann method (LBM) is a mesoscopic numerical simulation method between macro and micro. Compared with macro methods, it is more convenient for dealing with a complex boundary and, at the same time, it overcomes the limitations of the micro calculation method on size. It has the advantages of the automatic capture of a two-phase interface without manual processing or flexible boundary processing, and is suitable for parallel computing [24]. The lattice Boltzmann method has been widely used for simulating multiphase flow [25], micro-nano scale flow [26], turbulence [27], flow-induced vibrations [28], heat and mass transfer [29], and porous media flow problems [30,31]. Liu et al. [30] simulated the immiscible flow of wetting fluid and non-wetting fluid in two porous medias. Their simulation results confirmed that three different displacement modes are related to capillary number, viscosity ratio and the heterogeneity of porous media. Shi et al. [31] investigated the basic physical mechanism of Newtonian fluid replacing non-Newtonian fluid in porous media, which revealed the displacement mechanism of Newtonian fluid to non-Newtonian fluid from a mesoscopic perspective. Lautenschlaeger et al. [32] proposed a homogenization method for simulating multiphase flow in heterogeneous porous media, which is based on the lattice Boltzmann method and combines the gray level with the multi-component Shan-Chen method. This method makes the fluid–fluid and solid–fluid interactions in pores less than numerical discretization, and has been successfully applied to solving various single-phase and two-phase flow problems.

Unstable flows will create adverse effects for oil production, carbon dioxide storage and other aspects. In order to reveal the law of the unstable flow of fluid in porous media, some scholars have obtained the relevant phase diagrams through a large number of experiments. Some scholars have tried to improve the unstable flow of fluid by designing the structure of porous media. However, how a wetting gradient impacts viscous fingering remains unknown. In this paper, we used the lattice Boltzmann method to simulate the displacement process of fluids in porous media with a wetting gradient. The influence of the wetting gradient was studied over a wide range of capillary numbers (7.5 × 10^−6^ to 1.8 × 10^−4^), viscosity ratios (0.0025 to 0.04) and porosity (0.48 to 0.68). The flow results were quantitatively analyzed by means of quantitative parameters such as fractal dimension, displacement efficiency, saturation and interface length. Moreover, a phase diagram of flow stability results related to wetting gradient and capillary number was drawn.

## 2. Numerical Method

### 2.1. Mathematical Model

The lattice Boltzmann method is used for two-dimensional numerical simulation to simulate the displacement process of fluid in porous media. The lattice Boltzmann method is a mesoscopic simulation method between micro-molecular dynamics and macro-fluid dynamics. This method uses the Boltzmann transport equation to calculate two processes of collision and migration between micro-particles, replacing macro-particles with micro-particles. The macroscopic parameters of the system can be obtained by statistical averaging of a large number of particles without concern for the motion state of each particle. The motion behavior of the whole fluid is simulated and the corresponding macroscopic phenomena are analyzed. The Boltzmann transport equation is:

(1) $$f_{i}\left(r+e_{i},t+dt\right)=f_{i}(r,t)+\Omega_{i}\left(f_{0},\ldots ,f_{b}\right),i=0,\ldots ,b.$$

$f_{i}(r,t)$ is the distribution equation of particles in the direction *i* of position **r** at time *t*. $\Omega_{i}\left(f_{0},\ldots ,f_{b}\right)$ is collision operator.

Macro density ρ and velocity v on the node can be obtained by integration:

(2) $$\rho =\sum_{i=0}^{b}f_{i}$$

(3) $$\rho v=\sum_{i=0}^{b}f_{i}e_{i}.$$

**Figure 1:**
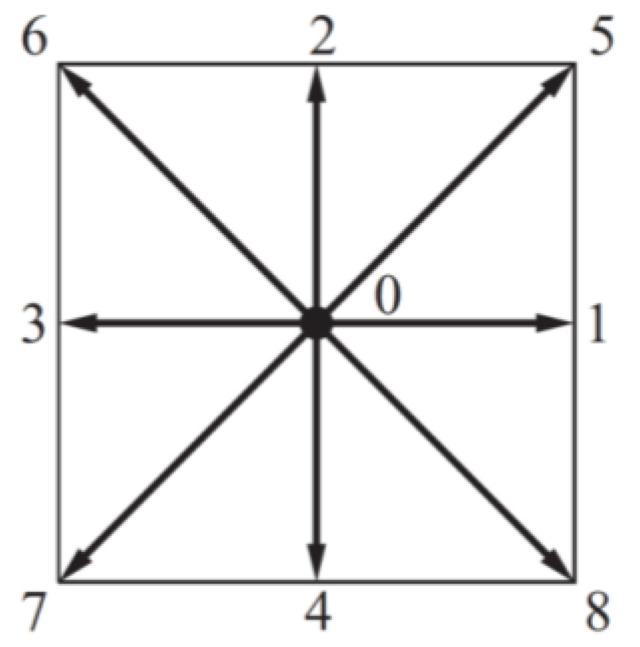
D2Q9 lattice model of velocities discretization.

The discrete velocities model adopted was the D2Q9 model, and its discrete velocities are shown in Figure 1.

The D2Q9 model illustrated in Figure 1 involves nine velocity vectors defined by:

(4) $${\vec{e}}_{i}=\left\{\begin{array}{ll} \left(0,0\right) & i=0\\ \left(1,0),(0,1),(-1,0),(0,-1\right) & i=1,2,3,4\\ \left(1,1),(-1,1),(-1,-1),(1,-1\right) & i=5,6,7,8 \end{array}\right.$$

The evolution equation of the particle distribution function of the multiple-relaxation time model is:

(5) $$f_{i}\left(r+e_{i},t+dt\right)=f_{i}(r,t)-M_{ij}^{-1}S_{ij}\left(\mu_{i}^{\mathrm{eq}}-\mu_{i}\right),$$

where $\mu_{i}=M_{ij}f_{j}$, $f_{j}$ is the distribution equation of the *j* direction and $\mu_{i}^{\mathrm{eq}}=M_{ij}f_{j}^{\mathrm{eq}}$, $f_{j}^{\mathrm{eq}}$ is the equilibrium distribution function in the *j* direction.

(6) $$\mu ={\left(\mu_{0},\mu_{1},\ldots ,\mu_{8}\right)}^{T}={\left(\rho ,e,\epsilon ,j_{x},q_{x},j_{y},q_{y},p_{xx},p_{yy}\right)}^{T},$$

where ρ is the density, e is the kinetic energy, ϵ is related to the kinetic energy square, $j_{x}$ and $j_{y}$ correspond to components of momentum, $q_{x}$ and $q_{y}$ correspond to the energy components, and $p_{xx}$ and $p_{yy}$ correspond to the symmetric traceless viscous stress tensors.

(7) $$\begin{array}{l} {\left.\rho \;\right|}_{i}={\left.e_{i}\right|}^{0}=1\\ {\left.e\;\right|}_{i}=-4{\left|e_{i}\right|}^{0}+3\left(e_{i,x}^{2}+e_{i,y}^{2}\right)\\ {\left.\epsilon \;\right|}_{i}=4{\left|e_{i}\right|}^{0}-21/2\left(e_{i,x}^{2}+e_{i,y}^{2}\right)+9/2{\left(e_{i,x}^{2}+e_{i,y}^{2}\right)}^{2}\\ {\left.j_{x}\right|}_{i}=e_{i,x}\\ {\left.q_{x}\right|}_{i}=e_{i,x}\left[-5{\left|e_{i}\right|}^{0}+3\left(e_{i,x}^{2}+e_{i,y}^{2}\right)\right]\\ {\left.j_{y}\right|}_{i}=e_{i,y}\\ {\left.q_{y}\right|}_{i}=e_{i,y}\left[-5{\left|e_{i}\right|}^{0}+3\left(e_{i,x}^{2}+e_{i,y}^{2}\right)\right]\\ {\left.p_{xx}\right|}_{i}=\left(e_{i,x}^{2}-e_{i,y}^{2}\right)\rho \\ {\left.p_{xy}\right|}_{i}=e_{i,x}e_{i,y}/\rho \end{array}.$$

Hence, the transformation matrix *M* is

(8) $$M=\left(\begin{array}{c} {\left.\rho \;\right|}_{i}\\ {\left.e\;\right|}_{i}\\ {\left.\epsilon \;\right|}_{i}\\ {\left.j_{x}\;\right|}_{i}\\ {\left.q_{x}\;\right|}_{i}\\ {\left.j_{y}\;\right|}_{i}\\ {\left.q_{y}\;\right|}_{i}\\ {\left.p_{xx}\;\right|}_{i}\\ {\left.p_{xy}\;\right|}_{i} \end{array}\right)=\left(\begin{array}{ccccccccc} 1 & 1 & 1 & 1 & 1 & 1 & 1 & 1 & 1\\ -4 & -1 & -1 & -1 & -1 & 2 & 2 & 2 & 2\\ 4 & -2 & -2 & -2 & -2 & 1 & 1 & 1 & 1\\ 0 & 1 & 0 & -1 & 0 & 1 & -1 & -1 & 1\\ 0 & -2 & 0 & 2 & 0 & 1 & -1 & -1 & 1\\ 0 & 0 & 1 & 0 & -1 & 1 & 1 & -1 & -1\\ 0 & 0 & -2 & 0 & 2 & 1 & 1 & -1 & -1\\ 0 & 1 & -1 & 1 & -1 & 0 & 0 & 0 & 0\\ 0 & 0 & 0 & 0 & 0 & 1 & -1 & 1 & -1 \end{array}\right).$$

$S_{ij}=\mathrm{diag}\left(s_{0},s_{1},s_{2},s_{3},s_{4},s_{5},s_{6},s_{7},s_{8}\right)$ is the diagonal relaxation matrix. In this approach, the kinematic viscosity υ and the bulk viscosity μ are related to the following relaxation parameters:

(9) $$\upsilon =c_{s}^{2}\left(\frac{1}{s_{7}}-\frac{1}{2}\right)=c_{s}^{2}\left(\frac{1}{s_{8}}-\frac{1}{2}\right)$$

(10) $$\mu =\frac{5-9c_{s}^{2}}{9}\left(\frac{1}{s_{1}}-\frac{1}{2}\right).$$

$c_{s}=c/\sqrt{3}$, $c_{s}$ is the sound velocity of the lattice; c=δx/δt, c is the lattice velocity; δx is the length step; and δt is the time step.

Applying the transformation matrix to the equilibrium probability distribution function, the raw moments at the equilibrium are

(11) $$\begin{array}{l} e^{eq}=-2\rho +3\left(j_{x}^{2}+j_{y}^{2}\right)\\ \epsilon^{eq}=\rho -3\left(j_{x}^{2}+j_{y}^{2}\right)\\ q_{x}^{eq}=-j_{x}\\ q_{y}^{eq}=-j_{y}\\ p_{xx}^{eq}=j_{x}^{2}-j_{y}^{2}\\ p_{xy}^{eq}=j_{x}j_{y} \end{array}.$$

The moments $\rho^{eq}$, $j_{x}^{eq}$ and $j_{y}^{eq}$ are not required as they will be multiplied by $s_{0}$, $s_{3}$, and $s_{5}$, which are zero.

**Figure 2:**
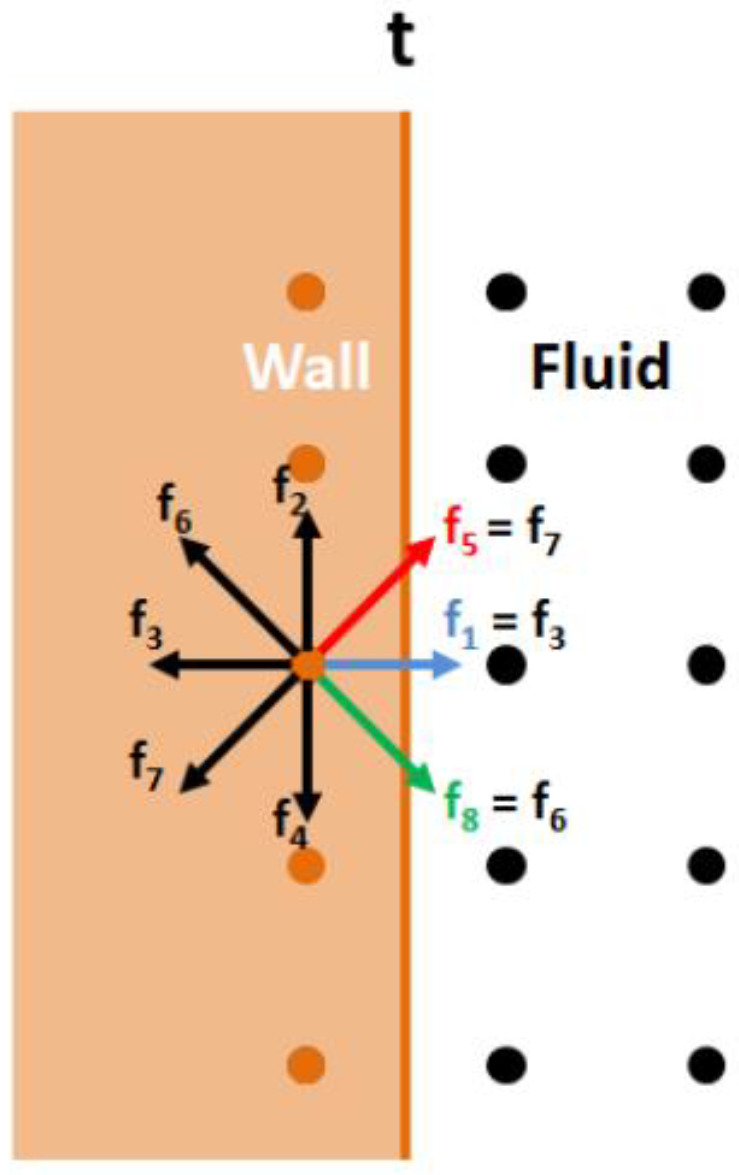
Bounce-back boundary condition.

The bounce-back boundary condition is used to implement the no-slip condition on a geometry wall. The incoming probability distribution functions at the wall node are reflected back to the initial fluid node, giving value to the unknown probability distribution functions. In the illustration in Figure 2, at a time *t*, the distribution function $f_{7}$ is reflected back to $f_{5}$, $f_{3}$ to $f_{1}$, and $f_{6}$ provides $f_{8}$ value.

The inlet and outlet boundary conditions used in the current simulations are velocity and pressure boundary conditions, respectively. The approach consists of the formulation of a linear system with mass and momentum conservation. This linear system will provide the value of the unknown probability distribution functions created after the streaming step as well as the value of ρ if a velocity boundary condition is imposed, or u if there is a pressure condition at the boundary. According to the Figure 2 example, after rearranging the moments in Equations (2) and (3) (density, x-momentum, y-momentum), the linear system can be written as:

(12) $$\begin{array}{l} f_{1}+f_{5}+f_{8}=\rho -\left(f_{0}+f_{2}+f_{4}+f_{3}+f_{6}+f_{7}\right)\\ f_{1}+f_{5}+f_{8}=\rho u+f_{3}+f_{6}+f_{7}\\ f_{5}-f_{8}=\rho v+f_{2}+f_{4}-f_{6}+f_{7} \end{array}.$$

A fourth equation is required to close the system and compute the value of velocity or pressure. A valid assumption is to apply the bounce-back rule for the nonequilibrium part of the particle distribution normal to the boundary. Therefore, considering that an inlet boundary is imposed on the left nodes of Figure 2, the fourth equation is

(13) $$f_{1}-f_{1}^{eq}=f_{3}-f_{3}^{eq}.$$

Solving the linear system, the unknown probability distribution functions are

(14) $$\begin{array}{l} f_{1}=\frac{2}{3}\rho u\\ f_{5}=f_{7}-\frac{1}{2}\left(f_{2}-f_{4}\right)+\frac{1}{6}\rho u+\frac{1}{2}\rho v\\ f_{8}=f_{6}-\frac{1}{2}\left(f_{2}-f_{4}\right)+\frac{1}{6}\rho u-\frac{1}{2}\rho v \end{array}.$$

For a velocity boundary condition, the linear system gives:

(15) $$\rho =\frac{1}{1-v}\left[f_{0}+f_{2}+f_{4}+2\left(f_{3}+f_{6}+f_{7}\right)\right].$$

For a pressure boundary condition, $p=\rho c_{s}^{2}$; taking v=0, the u is defined by:

(16) $$u=1-\frac{\left[f_{0}+f_{2}+f_{4}+2\left(f_{3}+f_{6}+f_{7}\right)\right]}{\rho }.$$

### 2.2. Simulation Setup

**Figure 3:**
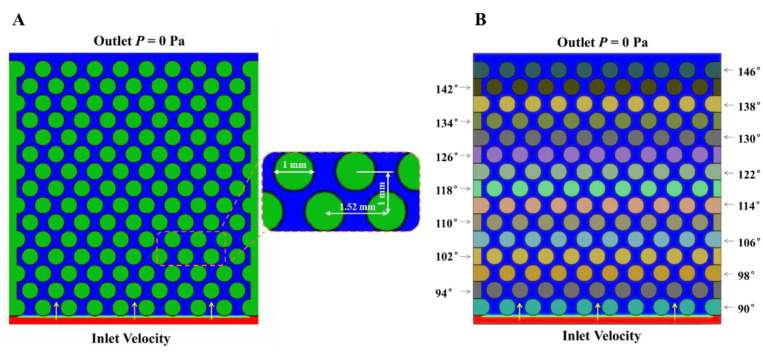
(**A**) Initial boundary conditions without wetting gradient. (**B**) Initial boundary conditions at the wetting gradient Δ*θ* = 4°. The red area is the displacing fluid, which is injected at a certain flow rate from below. The blue area is the displaced fluid, flowing out from the upper outlet. The outlet gauge pressure is 0 Pa. The horizontal line between the red area and the blue area represents the interface between the displacing fluid and the displaced fluid. The yellow arrow represents the flow direction. The green cylinder represents an impermeable solid material, while the area formed between the cylinder and the cylinder is a pore channel, and the fluid flows in the pore channel between the cylinders.

We used the computational fluid dynamics software XFlow to carry out the relevant numerical simulations. The phase field algorithm was employed for calculating the multiphase flow. The temperature type was set as isothermal. The computational domain and boundary conditions are shown in Figure 3. The size of the computational domain was 16 mm × 10 mm. The cylinders represent an impermeable solid material, while the area formed between the cylinders are the pore channels. The diameter of the cylinder and the spacing between cylinders are described in Figure 3A. The displaced fluid initially filled in the pores of the porous media, and the displaced fluid flowed in at a certain speed from the bottom, forcing the displaced fluid to flow out at the top. The initial gauge pressure field was 0 Pa (the corresponding actual pressure was 1.013 × 10^5^ Pa) and the initial velocity field was 0 m/s. The bounce-back boundary condition was used to implement the no-slip condition on the solid walls. The inlet and outlet boundary conditions used in the current numerical simulations were velocity and pressure boundary conditions. The displaced fluid was silicon oil (the wetting phase) and the displacing fluid was water (the non-wetting phase). The parameters of these two fluids were as follows: the density and viscosity of displacing fluid were set as *ρ*_1_ = 998.3 kg/m^3^, *μ*_1_ = 1 mPa s. The density and viscosity of the displaced fluid were set to *ρ*_2_ = 960 kg/m^3^, *μ*_2_ = 200 mPa s, and the viscosity ratio was *M* = 0.005. The surface tension *σ* = 28.2 mN/m. The inlet velocity was 0.001 m/s unless otherwise specified. The wetting gradient was set as follows: Take the wetting gradient Δ*θ* = 4° as an example, as shown in Figure 3B. The wetting angle of the lowest row of cylinders was set to 90°, and the same row of cylinders had the same wetting angle. The wetting angle was increased by 4° for each row up to the last row, thus creating the porous media of wetting gradient Δ*θ* = 4°. The wetting gradients Δ*θ* = 1°, Δ*θ* = 2° and Δ*θ* = 3° were arranged in the same way. The wetting gradient Δ*θ* = 0° meant that the overall wettability of the porous media was uniform and the overall wetting angle was 150°.

### 2.3. Model Verification

**Figure 4:**
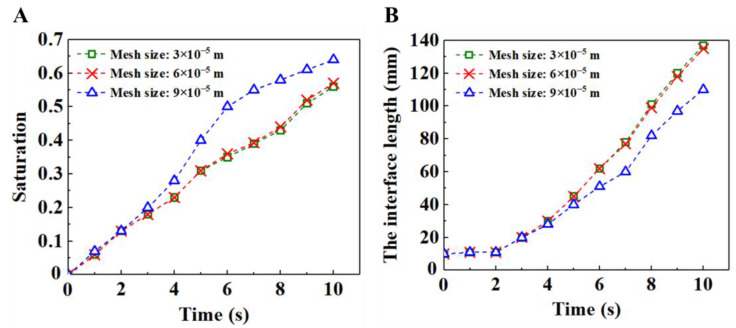
(**A**) Time evolution of the saturation of displacing fluid under different mesh sizes. (**B**) Time evolution of the interface length between the displacing fluid and the displaced fluid under different mesh sizes.

**Figure 5:**
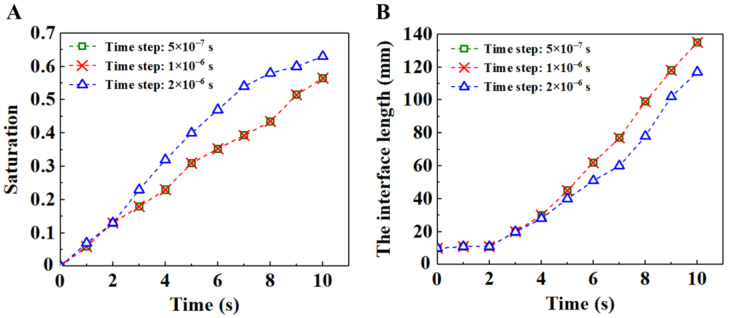
(**A**) Time evolution of the saturation of displacing fluid under different time steps. (**B**) Time evolution of the interface length between the displacing fluid and the displaced fluid under different time steps.

We first performed mesh independence verification and time step independence verification. The time steps selected for grid independence verification were all 1 × 10^−6^ s; the grid size selected for time step independence verification was 6 × 10^−5^ m. The total calculation time is the time required for the displacement fluid to flow to the porous media outlet. Figure 4 exhibits the time evolution of the saturation of displacing fluid and the interface length between the displacing fluid and the displaced fluid under different mesh sizes. The results show that the saturation and interface length tend to stabilize when the grid size is 6 × 10^−5^ m. Figure 5 presents the time evolution of the saturation of the displacing fluid and the interface length between the displacing fluid and the displaced fluid under different time steps. The results show that the saturation and interface length tend to be stable when the time step is 1 × 10^−6^ s. Thus, a grid size of 6 × 10^−5^ m and a time step of 1 × 10^−6^ s were employed in the subsequent numerical simulations.

**Figure 6:**
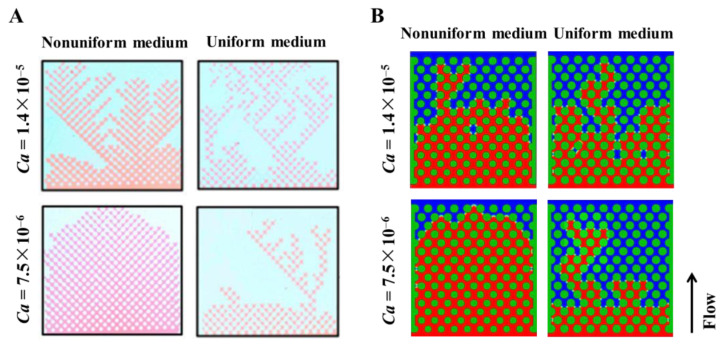
Results of the displacing fluid front morphologies at the time of the displacing fluid reaching the outlet under the condition of nonuniform medium and uniform medium with the capillary numbers of 7.5 × 10^−6^ and 1.4 × 10^−5^. (**A**) Experimental results of Rabbani et al. [11]. (**B**) Simulation results of the current investigation.

**Figure 7:**
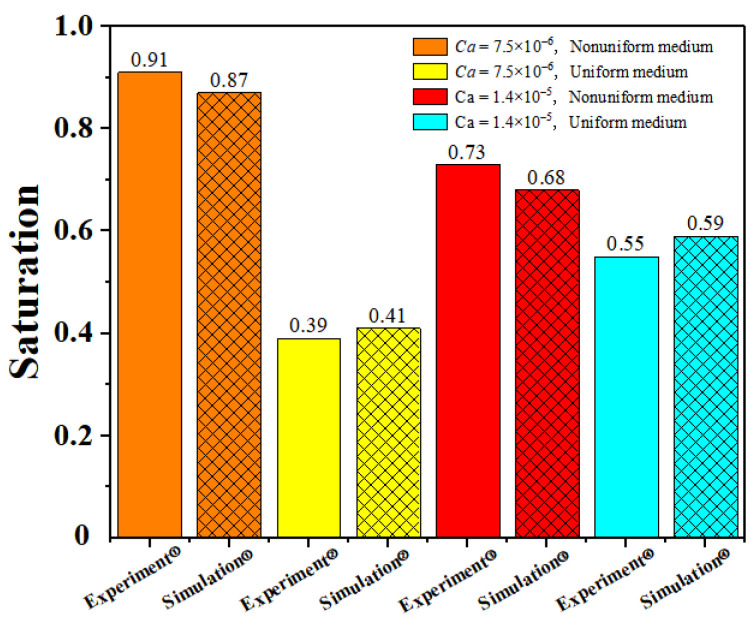
Comparison of the saturation between the simulation results and the experimental results under the condition of nonuniform medium and uniform medium with the capillary numbers of 7.5 × 10^−6^ and 1.4 × 10^−5^.

We selected the experimental results of Rabbani et al. [11] for experimental verification. Their experiment involves filling silicone oil into a porous medium arranged by cylinders. Then, water is injected into the porous medium to see how water displaces silicone oil in the porous medium. Among them, two different porous structures are used. One is the porous medium with uniform pores. The other is porous media with a pore gradient whose size increases from inlet to outlet. We selected four groups of experimental results to verify the current numerical model, including the fluid displacement processes in the porous mediums with and without pore gradient under the capillary number of 7.5 × 10^−6^ and 1.4 × 10^−5^. In order to save computing time and resources, we simulated a part of the experimental model. The fluid parameters and the amplitude of the pore gradient decline are consistent with the parameters in the experiment. The comparisons between simulation results and experimental results are shown in Figure 6 and Figure 7. The uniform medium shown in Figure 6 was composed of cylinders with the same diameter of 1 mm. The nonuniform medium in Figure 6 consisted of cylinders with the diameter decreasing row by row from the top to the bottom. The diameter difference between the adjacent row of cylinders was 0.0135 mm and the diameters of the cylinder in the top and bottom rows were 1 mm and 0.81 mm, respectively.

It can be seen from the comparison between the simulation results and the experimental results [8] that, when the capillary number is 7.5 × 10^−6^ in the nonuniform medium (the left figure of A and B in Figure 6), it can be simulated that the flow of displacing fluid in porous media is stable. In the other three cases, it can also be simulated that the displacing fluid has an unstable flow. In order to compare the simulation results with the experimental results more clearly, we also drew a diagram comparing the saturation under various conditions, as shown in Figure 6. It can be seen from the figure that the simulation results are similar to the experimental results, which shows that the lattice Boltzmann method can simulate fluid flow in porous media.

## 3. Results and Discussion

### 3.1. Effect of Wettability

**Figure 8:**
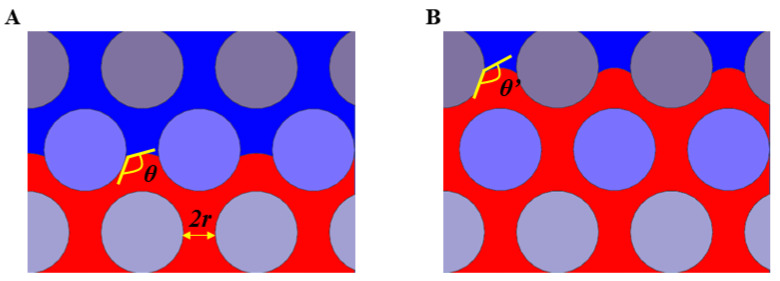
Schematic diagram of wetting angle change of a fluid in a porous medium with a wetting gradient. (**A**) The displacing fluid flows through a row of cylinders with a wetting angle of *θ*. (**B**) The displacing fluid flows through a row of cylinders with a wetting angle of *θ*’ (*θ*’ > *θ*). The displacing fluid is injected from the bottom upwards. The red and blue areas represent the displacing fluid and the displaced fluid, respectively. Cylinders with different colors represent the media with different wetting angles.

When the non-wetting fluid displaces the wetting fluid in a porous medium, it will flow forward only when the driving pressure of the displacement flow is greater than the capillary pressure threshold ~*σ*cos *θ*/*r*, where *r* is the pore radius. While the capillary pressure threshold can be adjusted by regulating the wettability to further regulate the fluid displacement process. It can be seen from the above formula that, with the increase of wetting angle, the capillary pressure threshold increases and then the flow resistance increases. Based on this, a porous medium with the wettability gradients will hopefully be designed to improve the flow stability, as shown in Figure 8. When the fluid flows from a row of cylinders with a wetting angle of *θ* to the next row of cylinders with a larger wetting angle of *θ*’, the flow resistance increases and the fluid will preferentially flow transversely and then forward.

**Figure 9:**
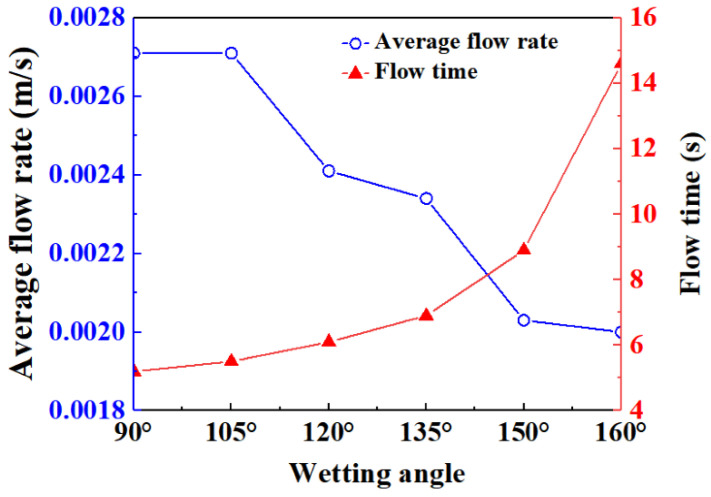
Variation curves of average flow rate and outflow time at different wetting angles.

In order to verify the influence of wettability on the fluid displacement process in the porous medium, the variation curves of average flow rate and outflow time at different wetting angles were investigated, as shown in Figure 9. With the increase in the wetting angle, the average flow rate of the displacement fluid decreases and the time required for the displacement fluid to flow out of the porous media increases. Thus, the flow resistance of the displacement fluid in porous media increases with the increment in wetting angle, which is consistent with the above theoretical analysis results.

### 3.2. Comparison between the Porous Media with and without Wetting Gradient

#### 3.2.1. Effect of Capillary Number

**Figure 10:**
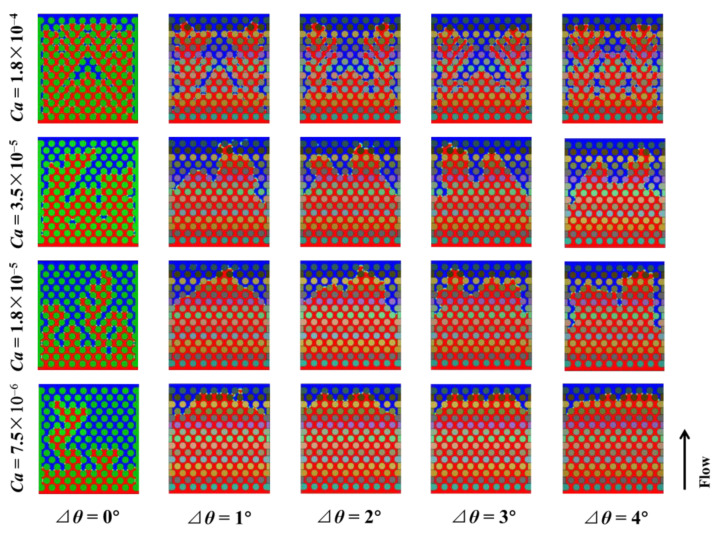
Simulation results of the displacing fluid front morphologies under different capillary numbers and wetting gradients at the time of the displacing fluid reaching the outlet. The viscosity ratio is 0.005.

Figure 10 shows the simulation results for different capillary numbers and wetting gradients. It can be seen that the flow pattern of the displacing fluid is unstable with or without wetting gradient when the capillary number is large. In the case of wetting gradient, the stability of the displacing fluid flow front is improved when the capillary number decreases. Without wetting gradient, the fluid still has more bifurcation. When the capillary number decreases to 7.5 × 10^−6^, the result with the wetting gradient is the best. The resistance gradient due to the wetting gradient can be fully displayed. In the absence of a wetting gradient, the flow of the displacing fluid is unstable even when the capillary number is reduced. It can be clearly seen from the simulation results in Figure 9 that the wetting gradient plays a significant role in improving the stability of the flow front. Furthermore, the smaller the capillary number (the lower the injection speed), the better the fluid filling, and the more stable the fluid front is.

**Figure 11:**
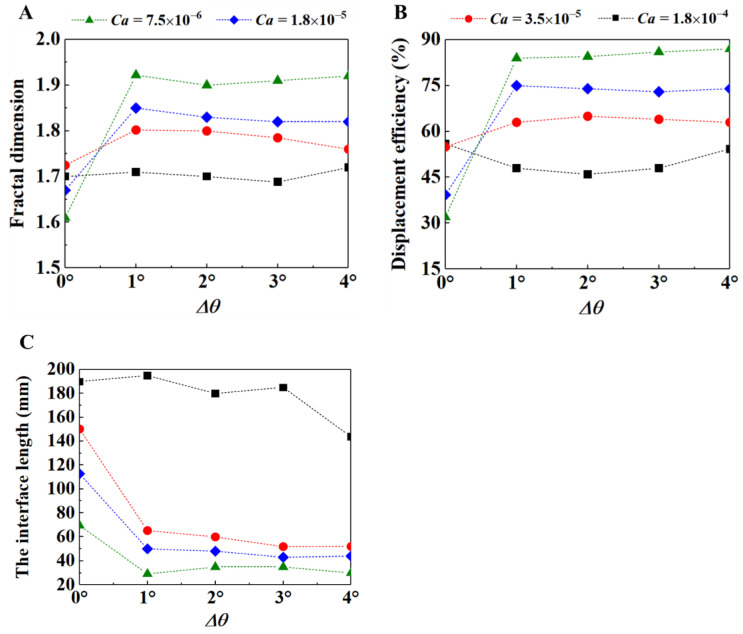
Effect of wetting gradient on the fractal dimension (**A**), the displacement efficiency (**B**), and the interface length (**C**) under different capillary numbers. The viscosity ratio is 0.005.

To quantitatively analyze the flow results shown in Figure 11, three parameters were selected for the quantitative analysis. The three quantitative parameters were the fractal dimension (Figure 11A), the displacement efficiency (Figure 11B), and the interface length (Figure 11C). It can be seen from the results of Figure 11A that, on the whole, the fractal dimension with the wetting gradient is larger than that without the wetting gradient. This indicates the effect of the wetting gradient on improving the flow stability of displacing fluids; with the wetting gradient, the capillary number is smaller and the fractal dimension is larger. The smaller the capillary number, the better the stability of the fluid front. This also indicates that both the wetting gradient and the capillary number affect the stability of the flow front. From the comparison of the displacement efficiency results of Figure 11B, it can be seen that, on the whole, the displacement efficiency with the wetting gradient is higher than that without the wetting gradient, which indicates that the existence of the wetting gradient improves the filling degree of the fluid. As the capillary number decreases, the displacement efficiency increases, and the filling degree improves. From the comparison of the results in Figure 11C, it can be seen that the interface length of porous media with the wetting gradient is much shorter than that without the wetting gradient, which also indicates that the bending degree of the fluid front interface is reduced. In addition, the smaller the capillary number, the shorter the interfacial length of the fluid front, the smaller the bending degree of the fluid front interface and the better the stability of the fluid front.

From the results of these quantitative analyses, when the capillary number increases to 1.8 × 10^−4^ under the condition of the wetting gradient, the overall flow stability is reduced and the filling effect is poor. This is because when the capillary number is 1.8 × 10^−4^, the corresponding inlet flow rate is relatively large, and the displacing fluid is subjected to a large force in the vertical direction, which exceeds the threshold range of the resistance gradient. The flow front of the displacing fluid is prone to local preferential flow and, when there is a wetting gradient, due to the effect of the resistance gradient, part of the displacement fluid flows laterally, making the bifurcation phenomenon of the displacement fluid front more serious. However, in the case of the wetting gradient, when the capillary number is less than 3.5 × 10^−5^, the overall filling effect is better and the stability of the flow is improved with the decrease of the capillary number. This is because the force of the displacement fluid in the vertical direction is less than the threshold range of the resistance gradient, which means the resistance gradient can inhibit the occurrence of local preferential flow.

**Figure 12:**
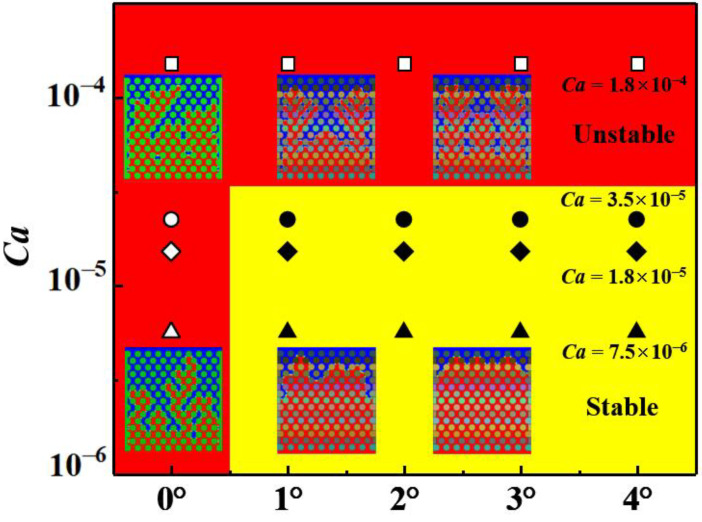
The phase diagram of the flow stability results related to wetting gradient and capillary number. The red area with solid symbols represents the unstable flow, and the yellow area with hollow symbols represents the steady flow. The viscosity ratio is 0.005.

We also plotted the phase diagram of the wetting gradient and capillary number, as shown in Figure 12. The identification of flow patterns in porous media can be considered comprehensively by way of parameters such as saturation, fractal dimension and finger width [33]. Therefore, we used this method to distinguish whether the flow of fluid in porous media is stable or unstable. It can be seen from the phase diagram that, in the case of the wetting gradient, the displacement process of fluid in porous media is mostly stable except for in the case of a large capillary number. The results of the fluid displacement process are unstable without the wetting gradient, which indicates that the wetting gradient plays a significant role in improving the flow stability of fluids in porous media.

**Figure 13:**
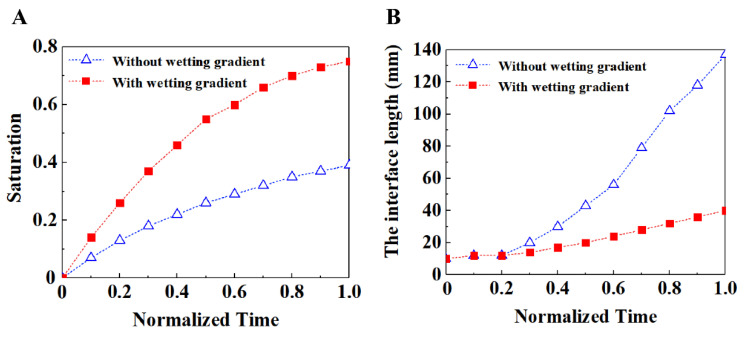
(**A**) Time evolution of the saturation of displacing fluid. (**B**) Time evolution of the interface length between the displacing fluid and the displaced fluid. In both cases, the capillary number is 1.8 × 10^−5^ and the viscosity ratio is 0.005. The solid and hollow symbols indicate the porous media with and without wetting gradient, respectively.

In order to better show the effect of wettability gradient on fluid flow in porous media, we selected a group of typical simulation results for comparison at different times. Figure 13 shows the comparison of saturation and interface length at different times with and without wetting gradient when the capillary number was 1.8 × 10^−5^. The time is normalized by the time required for the displacement fluid to flow to the porous media outlet, to characterize the different stages of the entire displacement process. The slope of the curve in Figure 13A shows that the filling speed of the fluid is faster when there is a wetting gradient, and the saturation result at any normalization time shows that the filling effect is better when there is a wetting gradient than when there is no wetting gradient. Figure 13B shows that the slope increases significantly in the case of no wetting gradient after the normalization time is 0.2, which indicates that there is unstable flow at the front of the fluid at this moment, especially the bifurcated finger-like flow, which makes the interface length of the front of the fluid significantly longer. In the case of the wetting gradient, the slope of the interface length is relatively flat, which means that the flow is relatively stable and there is no bifurcation phenomenon. So, the change of the interface length at the front of the fluid is not very large, which also means that porous media with a wetting gradient can better improve the stability of the flow front.

#### 3.2.2. Effect of Viscosity Ratio

**Figure 14:**
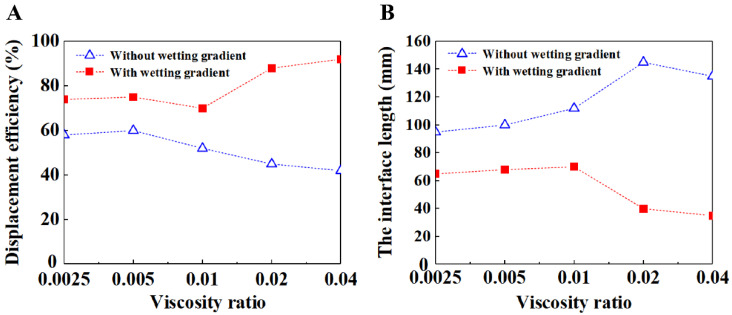
The effect of viscosity ratio on the displacement efficiency (**A**) and the interface length (**B**). In both cases, the capillary number is 3.5 × 10^−5^ and the porosity is 0.48. The solid and hollow symbols indicate the porous media with and without wetting gradient, respectively.

In order to clarify the influence of wetting gradient on the fluid displacement process with a wider range of parameters, we compared and studied the fluid displacement process of different fluid viscosities with and without a wetting gradient. We chose the case with a capillary number of 3.5 × 10^−5^ for our research. This is because under this capillary number, although there is a wetting gradient that plays a certain role in the stable flow of the fluid, there are still bifurcation phenomena at the front of the fluid, which affect the filling effect. Therefore, we tried to study how to improve the flow stability of the front of the fluid by changing the viscosity ratio. Figure 14A shows the comparison results of saturation with or without wetting gradient when the viscosity ratio is 0.0025, 0.005, 0.01, 0.02 and 0.04, respectively. We kept the viscosity of the displaced fluid unchanged at 1 mPa s, and changed the viscosity of the displaced fluid. The viscosities of the displaced fluid were set at 400 mPa s, 200 mPa s, 100 mPa s, 50 mPa s and 25 mPa s. From the comparison results in the figure, it can be seen that the change of viscosity ratio does have a great impact on the flow filling effect. On the whole, the saturation value under the condition of the wetting gradient is higher than that under the condition of no wetting gradient, which indicates that the filling effect under the condition of the wetting gradient is better. In addition, under the condition of the wetting gradient, the greater the viscosity ratio, the better the stability of the fluid front. Figure 14B shows the comparison results of interface length under different viscosity ratios. On the whole, the interface length under the condition of the wetting gradient is smaller than that under the condition of no wetting gradient, indicating that the flow stability under the condition of the wetting gradient is higher. The reason for this result is that when the viscosity ratio increases, the viscosity of the displaced fluid decreases, which makes the flow resistance of the injected fluid relatively lower; for the second half of the fluid flowing into the porous medium especially, the flow resistance decreases to a reasonable size, so that the effect of the resistance gradient can be reflected—the effect of the resistance gradient is to prevent the partial preferential breakthrough when the fluid flows in the porous medium. Therefore, due to the effect of the resistance gradient, the bifurcation phenomenon is reduced. In the case of the wetting gradient, the effect of the viscosity ratio on the wetting gradient is more obvious, so the flow of fluid in the porous media is more stable. In the case of no wetting gradient, the flow resistance of the corresponding injected fluid decreases due to the increase of viscosity ratio. At this time, there is no effect of resistance gradient, and the flow front is more prone to local breakthrough, leading to local preferential flow and affecting the stability of the displacement process.

**Figure 15:**
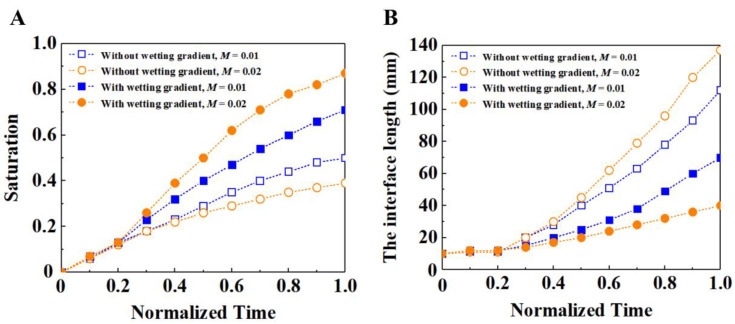
(**A**) Time evolution comparison of the saturation of displacing fluid under different viscosity ratios. (**B**) Time evolution comparison of the interface length between the displacing fluid and the displaced fluid under different viscosity ratios. In both cases, the capillary number is 3.5 × 10^−5^ and the porosity is 0.48. The solid and hollow symbols indicate the porous media with and without wetting gradient, respectively.

We selected two groups with a viscosity ratio *M* of 0.01 and 0.02 for comparative analysis at different times, as shown in Figure 15. On the whole, the saturation with a wetting gradient is higher than that without a wetting gradient, indicating that the filling effect is better with a wetting gradient. In terms of interface length, the interface length with the wetting gradient is smaller than that without the wetting gradient, which indicates that the stability of the fluid front is better with a wetting gradient; after the normalization time is 0.2, the interface length without the wetting gradient increases faster, which indicates that the unstable flow phenomenon begins at this time, and the finger-like flow becomes more and more obvious in the later stage, making the interface length increase faster. At the same time, the saturation of the fluid increases slowly. On the whole, the filling condition with the wetting gradient is better than that without a wetting gradient, and the filling effect with a high viscosity ratio is better than that with a low viscosity ratio.

#### 3.2.3. Effect of Porosity

**Figure 16:**
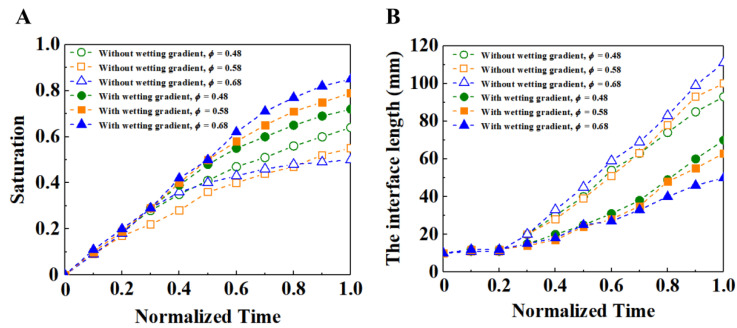
(**A**) Time evolution comparison of the saturation of displacing fluid under different porosities. (**B**) Time evolution comparison of the interface length between the displacing fluid and the displaced fluid under different porosities. In both cases, the capillary number is 3.5 × 10^−5^ and the viscosity ratio is 0.02. The solid and hollow symbols indicate the porous media with and without wetting gradient, respectively.

The size of the porosity also has a great impact on the flow of fluid in porous media., The size of porosity especially affects the function of the wetting gradient. Figure 16 shows the saturation and interface length of different porosities at the normalized time. It can be seen that, in the case of a wetting gradient, when the porosity is large, the slope of the fluid saturation is large, indicating that the flow front is relatively stable. This is because in the case of the wetting gradient, with the increase of porosity, the upper and lower spacings between cylinders become wider, and the contact area between fluid and porous media decreases. This can reduce the resistance gradient to an appropriate size, and then make the resistance gradient play a maximum role, which inhibits the occurrence of local breakthrough. On the whole, the filling condition with the wetting gradient is better than that without a wetting gradient, and the larger the porosity, the better the filling effect.

#### 3.2.4. Effect of Nonuniformity

**Figure 17:**
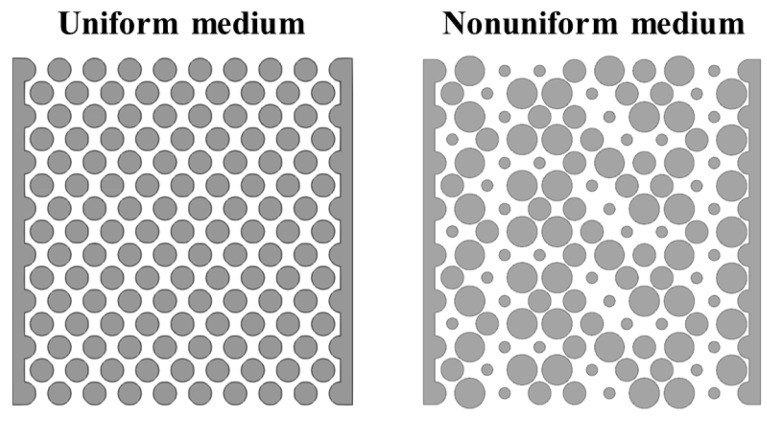
Schematic diagram of uniform and nonuniform medium, with the same porosity of 0.48. In order to maintain the same porosity as the uniform medium, the same number of cylinder diameters have been expanded and shrunk.

**Figure 18:**
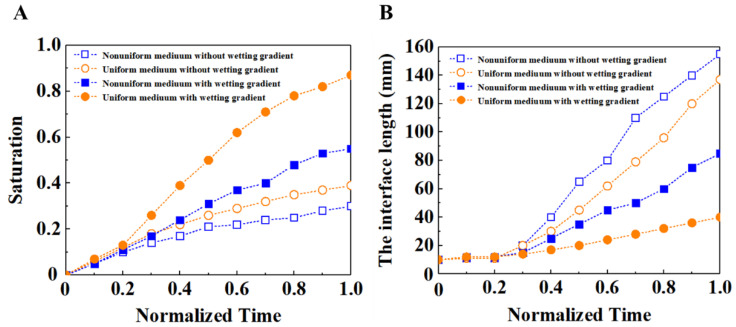
(**A**) Time evolution comparison of the saturation of displacing fluid in uniform medium and nonuniform medium. (**B**) Time evolution comparison of the interface length between the displacing fluid and the displaced fluid in uniform medium and nonuniform medium. In both cases, the capillary number is 3.5 × 10^−5^, the viscosity ratio is 0.02 and the porosity is 0.48. The solid and hollow symbols indicate the porous media with and without wetting gradient, respectively.

We randomly adjusted the diameter of some cylinders to obtain the nonuniform medium. In order to maintain the same porosity as the uniform medium, we adopted the method of expanding and shrinking the same number of cylinder diameters, respectively, to make the porosity of the nonuniform medium consistent with that of the uniform medium, as shown in Figure 17. In addition, we chose to compare the uniform media with a capillary number of 3.5 × 10^−5^, a viscosity ratio of 0.02 and a porosity of 0.48. From the comparison of saturation and interface length in Figure 18, it can be seen that under the condition of the wetting gradient, the flow stability of the displacement fluid in the nonuniform medium is indeed not as good as that in the uniform medium. This is because the nonuniformity of pores in the inhomogeneous medium causes the displacement fluid to flow preferentially in local areas, which makes the flow stability of the displacement fluid worse. In addition, under the condition of no wetting gradient, because there is no resistance gradient, this local preferential flow phenomenon is more likely to occur, resulting in the flow stability of the displacement fluid in the nonuniform medium being worse. However, from the comparison diagram, even in the case of nonuniform media, the filling effect of the displacement fluid with the wetting gradient is still better than that without a wetting gradient, which also shows the role of the wetting gradient in improving the stability of the displacement process.

## 4. Conclusions

In the present study, the displacement of immiscible fluid in porous media was numerically investigated using the lattice Boltzmann method. Our results demonstrate that the displacement pattern and efficiency can be controlled by the wetting gradient. The flow front stability can be improved by setting a wetting gradient on the porous media, which is confirmed over a wide parameter range of different wetting gradients, capillary numbers, viscosity ratios and porosities. When the capillary number is less than 3.5 × 10^−5^, the viscous fingering can be successfully suppressed and the transition from unstable to stable displacement can be achieved by the wetting gradient. While increasing the wetting gradient has little effect on the fluid displacement process, under the conditions of high viscosity ratio (*M* > 0.01) and large porosity (*Φ* > 0.58), the improvement of flow front stability is more significant when adding a wetting gradient. In addition, for the porous media with a wetting gradient, decreasing the capillary number, increasing the viscosity ratio or increasing porosity can improve flow front stability and filling efficiency, which is consistent with that of homogeneous wetting media. The present findings could be helpful for the design of porous products to suppress viscous fingering, which will be of great significance for industrial applications such as composite material preparation and oil exploration.

## Data Availability

Not applicable.
